# Bond Strength Evaluation of Ceramic Restorations with Immediate Dentin Sealing: A Systematic Review and Meta-Analysis

**DOI:** 10.30476/dentjods.2023.97057.1986

**Published:** 2024-09-01

**Authors:** Pooran Samimi, Pedram Iranmanesh, Maryam Khoroushi, Mohammad Hossein Kafi, Niloufar Jafari

**Affiliations:** 1 Dept. of Operative Dentistry, Dental Materials Research Center, Dental Research Institute, School of Dentistry, Isfahan University of Medical Sciences, Isfahan, Iran; 2 Dept. of Endodontics, Dental Research Center, Dental Research Institute, School of Dentistry, Isfahan University of Medical Sciences, Isfahan, Iran; 3 Dept. of Endodontics, School of Dentistry, Rafsanajn University of Medical Sciences, Rafsanjan, Iran; 4 Dept. of Operative Dentistry, School of Dentistry, Rafsanjan University of Medical Sciences, Rafsanjan, Iran

**Keywords:** Dentin, Bonding Agents, Resin cement, Shear Strength, Tensile Strength

## Abstract

**Statement of the Problem:**

Immediate dentin sealing (IDS) was introduced to overcome the disadvantages of delayed dentin sealing like pollution of dentin tubules, microleakage, and bond strength destruction over time. The effect of IDS on the bond strength of indirect restorations is still debatable.

**Purpose:**

This study was conducted to determine the effect of IDS on the bond strength of ceramic restorations to dentin

**Materials and Method:**

In this systematic review and meta-analysis, the study protocol was registered on the PROSPERO database under the registration number CRD420202014 27. MEDLINE (PubMed), Web of Science, Scopus, and ProQuest databases were searched until January 2021 and updated in January 2022. Worldcat.org and Opengrey.eu, ProQuest dissertation and thesis, and Google Scholar were searched to explore the grey literature. The *in vitro* studies evaluating the bond strength of ceramic restoration to dentin with and without IDS were included. Seven criteria were assessed to evaluate the risk of bias in the study. Statistical analyses were conducted using RevMan 5.3. The inverse variance method was used to determine the mean difference of micro-tensile bond strength (µTBS) and shear bond strength (SBS).

**Results:**

A total of 10 studies (20 datasets) were included in the meta-analysis. Regarding the µTBS analysis, IDS had a significantly higher bond strength than Delayed Dentin Sealing (DDS) (MD:1.16, 95%CI:0.28_2.03, I^2^=0%). However, no significant difference was found between them in the SBS analysis (MD:0.25, 95%CI: -0.56-1.06, I^2^=96%). All studies were categorized to have a moderate or high risk of bias.

**Conclusion:**

Most *in vitro* evidence showed favorable results for the effect of IDS on the bond strength and durability of indirect restorations. The adhesive system and the type of ceramic and its treatment before cementation are determining factors. Due to the heterogeneity of the outcomes and studies with a moderate/high risk of bias, the quality of the evidence was low.

## Introduction

The use of tooth-colored materials to restore decayed teeth, particularly in the posterior areas of the mouth, is important for many patients [ 1 ]. The direct use of composite resins for the reconstruction of teeth where gross tooth destruction has occurred and requires a vast reconstruction, especially the proximal contours, is too challenging and sometimes even impossible [ 2 ]. In these situations, the use of indirect restorations allows accurate reconstruction of the tooth crown and has better contours, wear resistance, and mechanical strength than the direct ones [ 2 - 3 ].

However, the disadvantages of indirect restorations such as tooth sensitivity that occasionally occurs in vital teeth, bond strength reduction over time, deboning, secondary caries, and fractures should be noted since they compromise the survival rate [ 4 - 5 ]. Hence, the improvement of bond strength is an important factor to enhance the success rate and fracture resistance, decrease microleakage, and increase the overall survival rate [ 6 ]. 

The conventional method used for the cementation of indirect restoration is delayed dentin sealing (DDS), which briefly includes the application of adhesive resins just before cementation [ 7 - 10 ]. Resin coating technique was introduced by Pashley *et al*. [ 11 ] in the 1990s to improve the properties of indirect restorations and to reduce tooth sensitivity. Later in 2005, Magne *et al*. [ 12 ] introduced immediate dentin sealing (IDS) based on the resin coating technique. This procedure involves the sealing of freshly cut dentin tubules filled with an adhesive resin alone or in combination with a low-viscosity resin prior to (digital or analog) impression-taking [ 12 ]. The use of IDS has been effective in improving the bond strength of indirect restorations [ 13 - 16 ]; however, some studies have indicated no priority for DDS regarding the long-term bond strength [ 17 ]. 

Considering the lack of consistency among the results of studies on the effect of IDS on the bond strength of indirect restorations and the lack of a comprehensive review in this field, this systematic review and meta-analysis aimed to evaluate the effect of IDS on the bond strength of ceramic restorations.

## Materials and Method

### Protocol and registration

The study protocol was registered on the PROSPERO database under the registration number CRD4202020-14 27. The Preferred Reporting Items for Systematic Reviews and Meta-Analyses (PRISMA) checklist was used to report this systematic review [ 18 ].

### Forming the question

The research question, based on the patient, intervention comparison, outcome, study (PICOS) framework, was “Does IDS improve the bond strength of ceramic restorations to dentin in comparison with IDS in experimental studies?”

The PICOS framework was set as P: human teeth with ceramic restoration, I: IDS, C: DDS, O: effect on micro-tensile bond strength (µTBS) or shear bond strength (SBS), and S: experimental studies.

### Study eligibility criteria

The inclusion criteria were all experimental studies that evaluated the SBS or µTBS of ceramic restorations to dentin using IDS. The exclusion criteria were studies that evaluated properties other than the bond strength of ceramic restorations to dentin, studies that evaluated the bond strength of other types of restorative materials, clinical trials, all types of reviews, case reports, or case series. 

### Information sources and search strategy

The keywords in the search strategy were defined based on the PICOS framework. An unlimited literature search was undertaken on the MEDLINE (PubMed), Web of Science, Scopus, and ProQuest databases. Worldcat.org and Opengrey.eu, ProQuest dissertation and thesis, and the first 100 results in Google Scholar were searched to explore the grey literature until January 2021 and updated in January 2022. A manual search was performed to explore the reference lists of all primary studies for the additional relevant publications linked to each primary study on the PubMed database. The search strategies in the four main databases are listed in Table 1. The search was restricted to English language.

**Table 1:** Search strategy of the databases from their foundation until January 2021 and updated January 2022

Database	Search line	Number of retrieved records
MEDLINE (PubMed)	(“immediate dentin sealing” OR “dentin sealing” OR “resin coating”) AND (“bond strength” OR “Shear bond” OR “tensile bond”)	78
Scopus	TITLE-ABS-KEY ((“bond strength” OR “Shear bond” OR “tensile bond”) AND (“immediate dentin sealing” OR “dentin sealing” OR “resin coating”))	94
Web of Science	TS= (((“immediate dentin sealing” OR “dentin sealing” OR “resin coating”)) AND (“bond strength” OR “Shear bond” OR “tensile bond”))	90
ProQuest	(“immediate dentin sealing” OR “dentin sealing” OR “resin coating”) AND (“bond strength” OR “Shear bond” OR “tensile bond”)	309

### Study selection and data collection

After the removal of the duplicate studies, the records were selected by titles and abstracts. In the next stage, full-text articles were screened for including records meeting the inclusion criteria. The study selection was done by two researchers independently, and any disagreement was resolved through discussion with other reviewers. Google sheets software was used as a customized extraction form to extract relevant data. The extraction form consisted of the first author’s name, year of publication, number of samples, adhesive system for IDS, type of aging protocol (mechanical or thermal or none), type of luting agent, type of ceramic, porcelain treatment before cementation, and main out come. Data were extracted by two reviewers independently, and any disagreement was resolved via discussion with other reviewers. In case of missing data, an email was sent to the corresponding author. If the authors did not answer up to one month twice, the record was excluded.

### Quality assessment

The quality assessment of each included study was independently assessed by two reviewers using the checklist of other systematic reviews [ 19 ]. The parameters consisted of (1) randomization of teeth, (2) use of teeth free of caries or restoration, (3) use of materials according to the manufacturer’s instructions, (4) use of teeth with similar dimensions, (5) description of sample size calculation, (6) treatment performed by the same operator, and (7) blinding of the operator of the testing machine. If it was possible to find the information in the article, it received an ‘‘Y’’ (yes) answer and vice versa. Studies that reported one to three items were classified as high risk of bias, four or five items as a medium risk of bias, and six or seven items as a low risk of bias. Any disagreements were resolved through discussion with a third reviewer.

### Synthesis of results

The data of each study were fed into RevMan 5.3 (The Cochrane Collaboration, Copenhagen, Denmark). Mean difference (MD) was determined for µTBS and SBS by inverse variance method. For subgroup analysis, studies were divided based on the bonding system used for the IDS, cement type, and ceramic restoration type and ceramic treatment before cementation. Statistical heterogeneity of the treatment effect was assessed using the inconsistency I^2^ test in which values greater than 75% were considered highly heterogeneous [ 20 ]. The sensitivity analysis was conducted by removing the studies with a high risk of bias. 

## Results

### Study selection

**Figure 1:**
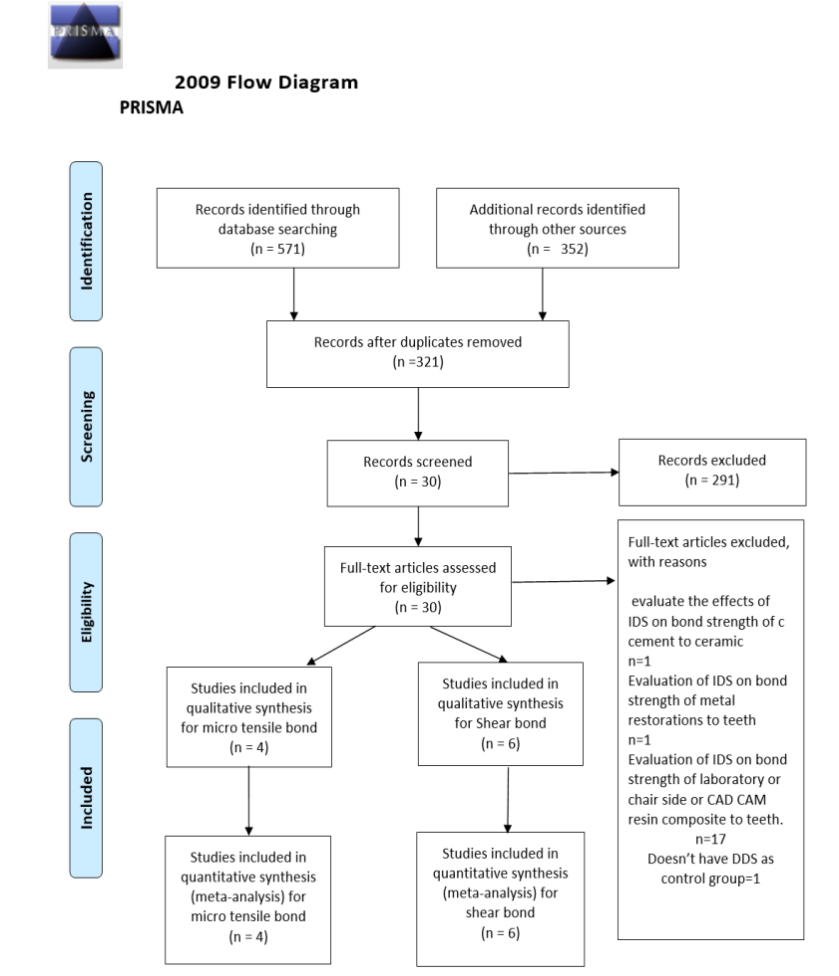
Preferred reporting items for systematic reviews and meta-analyses flow diagram of the search processes

A total of 892 relevant records were extracted from the databases. Figure 1 is a PRISMA flowchart that summarizes the article selection process. After the removal of duplicates, 321 records were evaluated for the titles and abstracts, from which 291 records were excluded. Therefore, 30 records were subjected to full-text evaluation. Of them, 20 studies were excluded. Table 2 shows the records excluded with reasons in the full-text assessment phase. Finally, 10 records [ 4 , 7 - 8 , 13 , 15 - 17 , 21 - 23 ] were used for qualitative and quantitative synthesis. Ten studies were included, of which 4 studies used µTBS test and 6 used SBS test.

**Table 2:** Studies excluded with reasons in the full-text asses-sment phase; IDS: immediate dentin sealing, DDS: delayed dentin sealing

Reasons for exclusion	References
Evaluation of the effects of IDS on the bond strength of cement to ceramic	n=1
Evaluation of the effect of IDS on the bond strength of metal restorations to teeth	n=1
Evaluation of the effect of IDS on the bond strength of laboratory or chair side or CAD CAM resin composite to teeth	n=17
Not having DDS as a control group	n=1

### Characteristics of the included datasets

As for µTBS test, all datasets used a non-self-adhesive luting system for the ceramic cementation and evaluated the bond strength of silica-based ceramics. Except one dataset, self-etch adhesive systems were used for IDS in other datasets [ 23 ] (Table 3). Regarding SBS test, all studies except one [ 17 ] used non-self-adhesive resin cement and all of the studies evaluated the bond strength of silica-based ceramics to teeth except one [ 13 ] which evaluated the bond strength of non-silica-based ceramics (monolithic zirconia). This study used two different materials for cementation (with Panavia F2 or PermaCem). It is noteworthy that only Panavia results were included for better comparison, the same as other included studies (Table 4). Each study in both categories used a different protocol for porcelain treatment before cementation, e.g. etching by Hydrofluoric acid (HF), Airborne-Particle Abrasion (APA), CoJet (CJ) abrasion, or none. There was a huge variation in the aging protocols in both categories.

**Table 3:** Studies that used µTSB

Study	Adhesive system	Sample size per group (N)	Type of aging	Type of luting agent	Type of ceramic	Porcelain treatment before cementation	IDS/ mean (Mpa) (SD)	DDS/ mean (Mpa) (SD)
Ishi *et al*. [ 21 ]	Etch-and-rinse	4	Artificial mechanical	Non-self-adhesive	Silica-based	Airborne Particle Abrasion (APA)	5.1(1.2)	3.5(1.6)
Hayashi *et al*. [ 16 ]	Self-etch	30	Artificial mechanical	Non-self-adhesive	Silica-based	Hydrofluoric acid (HF)	4.9(2.0)	3.8(1.7)
Kitayama* *et al*. [ 22 ]	Self-etch	14	Artificial mechanical	Non-self-adhesive	Silica-based	Airborne Particle Abrasion (APA)	12.97(5.82): N (60/98) (Number of beams, tested/ total)	– (0/89)
Kitayama. *et al*. [ 22 ]	Self-etch	14	No	Non-self-adhesive	Silica-based	Airborne Particle Abrasion (APA)	15.17 (5.24): N (49/81)	15.82 (4.22): N (45/78)
Murata**et al. [ 4 ]	Self-etch	8	Artificial mechanical	Non-self-adhesive	Silica-based	n/a	5.8(2.3)	4.4(1.5)

IDS: immediate dentin sealing, DDS: delayed dentin sealing, n/a: Not applicable, HF: Hydrofluoric acid, APA: Airborne particle abrasion, CJ: CoJet Superscript letters show different datasets from one study.

* Samples with artificial aging were not included because all samples in the DDS group failed in the pretest.

** Three different IDS applications were used, but just one of them was included in the analysis because this method was more similar to other included studies.

**Table 4:** Studies that used SBS test

Study	Adhesive System	Sample size pre group (n)	Type of aging	Type of luting agent	Type of ceramic	Porcelain treatment before cementation	IDS/ mean (Mpa) (SD)	DDS/ mean (Mpa) (SD)
Falkensammer *et al*. [ 7 ]	Self-etch	48	No	Non-self-adhesive	Silica-based	Hydrofluoric acid (HF)	13.7(4.7)	19.5(4.0)
Shakal*et al. [ 8 ]	Etch-and-rinse	10	Artificial thermal	Non-self-adhesive	Silica-based	Airborne Particle Abrasion (APA)	7.50(0.78)	8.00(0.31)
Shakal *et al*. [ 8 ]	Etch-and-rinse	10	No	Non-self-adhesive	Silica-based	Airborne Particle Abrasion (APA)	9.42(0.56)	10.06(0.44)
Shakal *et al*. [ 8 ]	Etch-and-rinse	10	Artificial thermal aging	Non-self-adhesive	Silica-based	Hydrofluoric acid (HF)	8.00(0.79)	8.82(0.389)
Shakal *et al*. [ 8 ]	Etch-and-rinse	10	No	Non-self-adhesive	Silica-based	Hydrofluoric acid (HF)	8.14(0.44)	8.86(0.384)
Shakal *et al*. [ 8 ]	Etch-and-rinse	10	Artificial thermal aging	Non-self-adhesive	Silica-based	CoJet (CJ)	7.62(0.49)	8.10(0.22)
Shakal *et al*. [ 8 ]	Etch-and-rinse	10	No	Non-self-adhesive	Silica-based	CoJet (CJ)	8.14(0.44)	10.50(0.41)
Shakal *et al*. [ 8 ]	Etch-and-rinse	10	Artificial thermal aging	Non-self-adhesive	Silica-based	N	4.44(0.52)	4.88(0.544)
Shakal *et al*. [ 8 ]	Etch-and-rinse	10	No	Non-self-adhesive	Silica-based	N	6.00(0.79)	7.52(0.37)
Reboul *et al*. [ 23 ]	Etch-and-rinse	10	No	Non-self-adhesive	Silica-based	Hydrofluoric acid (HF)	15.74 (2.12)	12.07(1.41)
Choi**et al. [ 15 ]	Etch-and-rinse	10	Artificial thermal aging	Non-self-adhesive	Silica base	Hydrofluoric acid (HF)	4.11(2.82)	3.14(1.47)
Choi *et al*. [ 15 ]	Self-etch	10	Artificial thermal aging	Non-self-adhesive	Silica-bases	Hydrofluoric acid (HF)	11.18 (4.75)	3.14 (1.47)
Dalby*** *et al*. [ 17 ]	Etch-and-rinse	13=Opti bond FL	No	Self-adhesive	Silica-based	Hydrofluoric acid (HF)	10.03 (3.50)	7.17(2.09)
		11=single bond					8.24(3.35)	
		11=DDS						
Dalby *et al*. [ 15 ]	Self-etch	8 samples=Go!	No	Self-adhesive	Silica-based	Hydrofluoric acid (HF)	6.94 (1.53)	7.17(2.09)
		11 samples= one coat bond						
		11 samples in DDS group					7.21(2.83)	
Rigos ****et al. [ 13 ]	Etch-and-rinse	15	No	Non-self-adhesive	Non-silica-based	Airborne Particle Abrasion (APA)	39.94 (1.34)	33.40 (1.76)
Rigos *et al*. [ 13 ]	Etch-and-rinse	15	No	Non-self-adhesive	Non-silica-based	CoJet (CJ)	38.68 )1.16)	29.37(2.16)

* According to different porcelain treatment before cementation and aging or non-aging variables included in eight datasets

**,*** Each included two datasets according to different adhesive systems.

**** Assessed in two data sets according to different porcelain treatment before cementation

### Risk of bias in individual studies

Overall, in the µTBS group, all studies were categorized as moderate (1 study; 25%) or high risk of bias (3 studies; 75%). In the SBS group, four (66.6%) and two (33.3%) studies showed a moderate and high risk of bias, respectively. No studies (100%) in each group mentioned sample size calculation and blinding of the operator of the testing machine. Moreover, 100% and 66.6% of studies in the µTBS and SBS groups did not mention the treatment was performed by a single operator. Hence, at least three out of seven items received NO answers for included studies, and none of them was categorized as a low risk of bias (Tables 5 and 6).

**Table 5:** Assessment of the risk of bias of µTSB (n = 4)

Study	Tooth randomization	Teeth free of caries or restoration	Materials used according to the manufacturer’s instructions	Teeth with similar dimensions	Sample size calculation	Treatment performed by a single operator	Blinding of the operator of the testing machine	Risk of bias
Maeno *et al*. [ 16 ]	N	Y	Y	Y	N	N	N	High risk
Kitayama *et al*.[ 22 ]	Y	Y	Y	Y	N	N	N	Moderate risk
Maeski *et al*.[ 4 ]	N	Y	Y	Y	N	N	N	High risk
Ishi *et al*.[ 21 ]	N	Y	Y	Y	N	N	N	High risk

**Table 6:** Assessment of the risk of bias of shear bond strength (SBS) (n = 6)

Study	Tooth randomization	Teeth free of caries or restoration	Materials used According to the manufacturer’s instructions	Teeth with similar dimensions	Sample size calculation	Treatment performed by a single operator	Blinding of the operator of the testing machine	Risk of bias
Falkensammer *et al*.[ 7 ]	No	Yes	Yes	Yes	No	No	No	High risk
Choi *et al*. [ 15 ]	Yes	Yes	Yes	Yes	No	No	No	Moderate risk
Dalby *et al*. [ 17 ]	Yes	Yes	Yes	Yes	No	Yes	No	Moderate risk
Shakal *et al*. [ 8 ]	Yes	Yes	Yes	N	No	No	No	High risk
Reboul *et al*. [ 23 ]	Yes	Yes	Yes	Yes	No	Yes	No	Moderate risk
Rigos *et al*. [ 13 ]	Yes	Yes	Yes	Yes	No	No	No	Moderate risk

“Y” = “Yes” and shows reported item “N” = “No” and shows not reported item

### Meta-analysis

**Figure 2:**
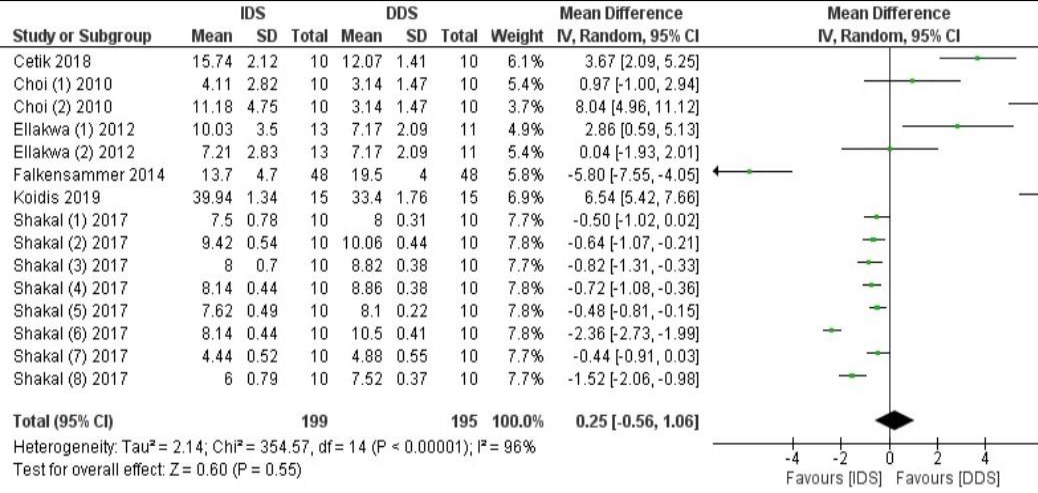
Forest plot of the analysis of immediate dentin sealing (IDS) on shear bond strength (SBS) compared to Delayed Dentin Sealing (DDS). Event: shear bond strength (SBS) in Mpa

The meta-analysis indicated that IDS had no positive effect on the SBS (MD:0.25, 95%CI: -0.56-1.06, I^2^= 96%) (Figure 2). In subgroup analysis, IDS demonstrated no positive effect on the SBS when silica-based ceramics were used (MD: -0.36, 95%CI: -1.00-0.27, I^2^=93%). Li-kewise, in subgroup analysis, IDS demonstrated no positive effect on the SBS when non-self-adhesive cements were used as a luting agent (MD: 0.12, 95%CI: -0.73-0.96, I^2^=97%). The results of the analysis of non-artificial and artificial aging datasets showed no statistically significant difference (MD: -0.12, 95%CI: -1.64-1.40, I^2^=98% and MD: -0.10, 95% CI: -0.77-0.57, I^2^=85%), respectively). Although applying etch-and-rinse systems for IDS did not improve the SBS (MD:1.06, 95%CI: 0.36-2.09, I^2^=98%), the self-etch systems enhanced the SBS (MD:0.66, 95% CI:-6.38-7.69), I^2^=97%).

**Figure 3:**
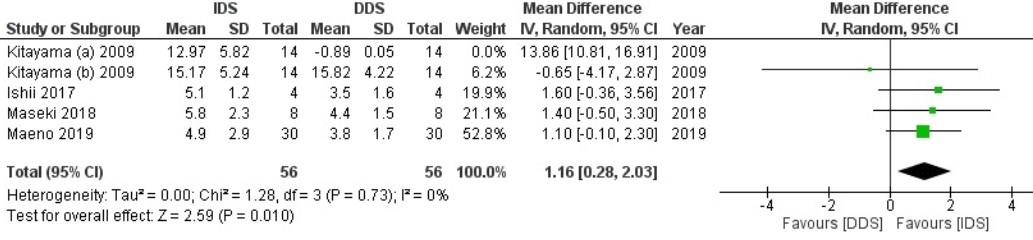
Forest plot of the analysis of immediate dentin sealing (IDS) on µTBS compared to delayed dentin sealing (DDS). Event: µTBS in Mpa

The meta-analysis indicated that IDS had a positive effect on the µTBS (MD:1.16, 95%CI:0.28_2.03, I^2^= 0%) (Figure 3). In subgroup analysis, IDS improved the µTBS after aging or applying self-etch adhesive systems (MD:1.27, 95%CI:0.37-2.18, I^2^=0% and MD: 1.04, 95%CI: 0.07-2.05, I^2^=0%, respectively).

The results of sensitivity analysis (after eliminating the high-risk studies) in both SBS and µTBS categories showed that MD was significantly higher in DDS than in IDS (MD:4.13 95%CI:078-7.48, I^2^=96.5% and MD:-0.65 95%CI:-4.17-2.78, I^2^=NA, respectively), which was different from the main analysis. Hence, the robustness of the analysis was low.

## Discussion

IDS was first introduced in 2005 to improve adhesion and restorative adaptation and to protect the pulp vitality [ 24 ]. The primary technique involves the application of an etch-and-rinse adhesive to the dentin surface. After taking the impression in the delivery session, an indirect restoration was applied after sandblasting with a non-self-adhesive cement on the IDS surface, which was conditioned by a brush and pumice [ 12 ]. Since then, different IDS methods with different types of bonding, intaglio surface preparation methods, IDS surface conditioning, and direct and indirect restorations with different materials have been studied, which have shown different results [ 25 - 28 ]. Therefore, the outcome of the present study may help practitioners make better clinical decisions.

The results of the included studies according to the test used for measuring bond strength were assessed in two categories: SBS and µTBS. The present meta-analysis showed that µTBS was higher with IDS than with DDS, and there was no significant difference between the two groups in SBS. The SBS has easy sample preparation and less technical sensitivity, but the samples in µTBS can be highly affected by adverse events such as premature failures and need a larger number of samples [ 25 - 28 ]. Yet, the more uniform stress distribution achieved by the µTBS test than the SBS [ 29 ] is a considerable factor, so the simplicity of the SBS test seems not to be a good reason for choosing this test to evaluate the bond strength [ 30 ]. 

In case of mechanical or thermal aging of the samples, the µTBS of IDS was higher than that of DDS, but the difference was not significant in the SBS analysis. Thus, the use of IDS may increase the bonding durability. The positive effect of IDS on durability is probably due to the sufficient and effective penetration of the resin into the newly cut dentin collagen fibers of the tooth and the formation of a sufficiently thick hybrid layer compared to DDS. In the absence of dentin sealing, the collagen fibers collapse during impression-taking, and their interfibrillar spaces are reduced for the resin to penetrate [ 31 - 32 ]. On the other hand, in the case of temporary restoration, even despite the use of various surface cleaning methods to remove cement residues (such as air-abrasion), dentin is contaminated with temporary cement and prevents adequate interaction between the adhesive and collagen [ 33 ]. If temporary restoration is not used, or restoration does not have enough sealing, the dentin becomes contaminated, and all these factors interfere with the penetration of the resin and the formation of an effective hybrid layer, which endangers the immediate bond strength of the dentin [ 34 ]. 

IDS also prevents the denaturation of collagen structure over time by sealing tubules and preserving exposed collagen in the freshly cut dentin and preventing the contamination and activation of proteolytic enzymes [ 35 , 36 ]. Furthermore, this layer acts as a stress reliever and protects the bonding layer against mechanical forces [ 4 ]. Another positive effect is due to the maturation of the adhesive layer (IDS) by the dark curing mechanism and the continuation of polymerization until the cementation is performed. This process reduces the stresses due to the polymerization of the cement and the occlusal forces on the newly created hybrid layer with low strength compared to DDS [ 37 - 38 ]. Since the bond strength decreases over time, according to the results, it may be possible to confirm the results of previous studies about the effect of IDS on increasing the bond durability [ 12 , 16 ].

The bond strength, in terms of the type of adhesive system, indicated that although the SBS of IDS was not higher in the etch-and-rinse system, the µTBS of IDS was higher in the self-etch subgroup. The bonding systems with fillers or functional monomers (creating a chemical bond) in the etch-and-rinse subgroup of the SBS group were used in most studies [ 39 - 41 ], which, if used correctly, create high strength. Thus, IDS can be used with all types of bonding systems to create sufficient film thickness as recommended by Magne *et al*. [ 42 ]. It can also be as effective as OptiBond FL, which is the gold standard of adhesive materials.

The µTBS and SBS of IDS and DDS groups were the same when different ceramic surface preparation methods were applied before cementing the restoration. However, the bond strength decreased in preparation with silicate, air-abrasion, and hydrofluoric acid, respectively. This result can be due to the dual chemical and mechanical bonding properties of air-abrasion systems with silicate particles and increased surface roughness in air-abrasion compared to hydrofluoric acid [ 8 , 43 - 44 ]. Therefore, surface preparation methods are highly effective in improving the bond strength, and the main purpose of using IDS is not to increase the bond strength.

Despite the numerous advantages mentioned for IDS in studies, this technique is time-consuming and requires more materials and steps. This method has a high technical sensitivity. If the adhesive layer is too thick, the strength of ceramic restorations will decrease due to less space and a large difference in the elastic coefficient of the adhesive layer and restorations, especially ceramic restorations [ 45 - 46 ]. On the other hand, if a very thin adhesive layer (less than 40 microns) is formed, all the thickness of the adhesive turns into an air-inhibited layer and the adhesive does not polymerize, and this method practically loses its clinical effectiveness [ 47 ]. To reduce the interference of temporary restorations and common impression materials, the tooth should be covered with Vaseline after IDS so that the monomers in the temporary restorative resin are not bonded to the adhesive layer [ 24 , 48 ]. Moreover, instead of using temporary cement, mechanical gear should be created with the help of undercuts, embracers, and temporary splints, and the final restoration should be delivered and cemented in the shortest possible time (up to 1 week) [ 49 ]. To eliminate the interference with the impression materials, it is recommended to use digital impression-taking methods, and if impression materials are used, the oxygen-inhibiting layer of the IDS surface should be thoroughly cleaned and removed to prevent complete polymerization of the impression materials [ 50 - 51 ].

Few clinical studies have investigated the effectiveness of this technique. Gresnigt *et al*. [ 52 ] reported that IDS increased the survival rate of restorations if more than 50% of dentin was exposed. However, Van den Breemer *et al*. [ 53 ] showed IDS was not superior in the survival rate and success of restorations. The heterogeneity of the population in terms of oral health and different experiences of clinicians may be the reasons for these contradictions [ 53 ].

Due to the lack of clinical trials, the present study was performed on *in vitro* studies. The high heterogeneity of the SBS studies indicates the diversity of the materials and methods used. The quality of most studies in both groups was categorized as moderate to high risk of bias. It should be noted among a seven-item criterion proposed, three criteria of sample size calculation, the blinding of the operator of the testing machine, and performing the treatment by one operator were not mentioned in most studies. Thus, several variables in the design of laboratory studies were not controlled or reported, which might be due to the lack of an accepted guideline. By excluding the high risk of bias studies, the outcome was different from the main outcome for each group, indicating low consistency. Most of the included studies evaluated the SBS. However, owing to the advantages of µTBS, it is suggested for the bond strength evaluation in this area. More studies with a better design are needed to achieve a definitive result. 

## Conclusion

Most *in vitro* evidence showed the favorable impact of IDS on the bond strength and durability of indirect restorations. The use of any standard etch-and-rinse adhesive system or self-etching system is effective to obtain the desired results with IDS. The use of pre-treatment ceramic surface preparation methods reduces the difference in the IDS impact. However, the results of the *in vitro* studies should be used in clinical settings with caution. In addition, the included studies have low-quality evidence, so more high-quality research is needed.
